# n-Butanol Extract of *Polygonum capitatum* Targets Biofilm Formation, Motility, and Adhesion Attenuation to Combat Uropathogenic *Escherichia coli*

**DOI:** 10.3390/cimb48030265

**Published:** 2026-03-02

**Authors:** Derong Zeng, Yan Zhang, Jingjing Guo, Jiahua Yu, Shuai Dou, Yuqi Yang, Xiang Yu, Yongqiang Zhou, Juan Xue, Zehuan Wang, Wude Yang

**Affiliations:** 1 College of Pharmacy, Guizhou University of Traditional Chinese Medicine, Guiyang 550025, China; 2 Centre in Artificial Intelligence Driven Drug Discovery, Faculty of Applied Sciences, Macao Polytechnic University, Macao, China; 3 School of Basic Medicine, Guizhou University of Traditional Chinese Medicine, Guiyang 550025, China

**Keywords:** *Polygonum capitatum*, UTI, UPEC, biofilm formation, motility, adhesion and invasion

## Abstract

Uropathogenic *Escherichia coli* (UPEC) that form biofilms exhibit high-level antibiotic resistance, which poses substantial challenges to current therapeutic strategies for urinary tract infection (UTI). There is an urgent need for strategies specifically targeting UPEC biofilms. This study investigated the effects of the n-butanol extract of *Polygonum capitatum* (BPC) on UPEC strains, focusing on its antibacterial activity, biofilm formation, bacterial motility, adhesion capacity, and cell membrane integrity. The disk diffusion method, minimum inhibitory concentration (MIC), and minimum bactericidal concentration (MBC) assays demonstrated that BPC exhibited potent antibacterial activity against both reference and clinically isolated UPEC strains. Time–kill curve assays further confirmed that BPC inhibits bacterial growth in a time-dependent manner. BPC inhibited UPEC biofilm formation in a dose-dependent manner, significantly reducing biofilm formation in both reference and clinical UPEC strains. Furthermore, BPC disrupted cell membrane integrity in UPEC strain CFT073, resulting in the leakage of alkaline phosphatase (AKP), β-galactosidase, and intracellular proteins. BPC treatment also significantly reduced bacterial surface hydrophobicity, impaired swimming and swarming motility, and diminished adhesion and invasion capabilities. A total of 32 active compounds, predominantly flavonoids, were identified in BPC by UHPLC-Q-orbitrap MS/MS. Molecular docking studies revealed that several compounds in BPC, such as quercetin-3,4′-O-di-beta-glucoside, exhibited strong binding affinity to AKP and β-galactosidase, further supporting its potential to disrupt membrane integrity and inhibit biofilm formation. Thus, BPC exerts anti-UPEC effects through biofilm disruption and multi-targeted anti-virulence mechanisms, highlighting its potential as a novel therapeutic or adjunctive agent for UTI, particularly against recalcitrant biofilm-associated infections. The mode of action of BPC provides a scientific basis for developing new anti-infective strategies as alternatives to conventional antibiotics.

## 1. Introduction

Urinary tract infection (UTI) refers to an inflammatory response caused by the invasion of various pathogens into the urothelium, with approximately 150 million cases reported annually [1,2]. Notably, up to 50% of women and 12% of men will be afflicted by a UTI in their lifetime, with 20% to 44% of affected women experiencing recurrent infections [3,4]. Uropathogenic *Escherichia coli* (UPEC) is the predominant causative pathogen, accounting for 70% to 95% of community-acquired UTIs and approximately 50% of nosocomial cases, imposing a substantial burden of morbidity and mortality [5,6]. Currently, the overuse of antibiotics and the prophylactic treatment of UTI have led to the development of multidrug resistance in UPEC, thereby significantly complicating therapeutic management [7]. A bacterial biofilm is a structured microbial community embedded within a self-produced matrix of extracellular polymeric substances (EPS) and attached to a surface. Biofilms represent a primary source of numerous serious infections, including those caused by multidrug-resistant, broad-spectrum drug-resistant, and complete drug-resistant bacteria. Studies have indicated that up to 80% of all bacterial infections are associated with biofilm formation [8,9]. The biofilm-forming capacity of UPEC plays a critical role in the development of persistent and recurrent UTI [10]. During colonization of the urinary tract, UPEC utilizes mechanisms such as the invasion of urothelial cells and biofilm formation to evade elimination by the host immune system and thereby develop antibiotic resistance [11]. Furthermore, the adhesive capability and motility of UPEC are critical factors influencing its virulence expression and pathogenic process [12]. UPEC utilizes motility to evade the host immune system and migrate to new sites of infection within the bladder lumen or ascend to the kidneys [13]. Concurrently, bacterial motility facilitates the ascent of UPEC to the upper urinary tract and subsequent dissemination into the bloodstream, thereby contributing to the establishment of persistent infections [14]. The adhesion and colonization of UPEC on uroepithelial cells constitute the critical initial step that triggers the UTI cascade and promotes successful infection establishment [15,16]. Therefore, developing targeted therapeutic interventions aimed at disrupting or inhibiting UPEC biofilms is crucial to reduce bacterial adhesion, invasion and overcome antibiotic resistance.

Traditional Chinese Medicine (TCM) demonstrates unique advantages in preventing and treating UTI, characterized by its multi-component nature, multi-target efficacy, multi-pathway mechanisms, and low toxicity with minimal side effects, making it a valuable source of inspiration for novel therapeutic agents [17]. The traditional Chinese herb *Polygonum capitatum* is employed to treat various urinary system disorders, such as pyelonephritis and UTI. Its aqueous extract serves as the primary ingredient in Sijicao Granules and Relinqing Granules, the latter of which was adopted as a clinical treatment for COVID-19 infectious pneumonia in Guizhou Province [18,19,20]. The primary active constituents of *Polygonum capitatum* have been reported to be flavonoids and phenolic acids, which exhibit a range of pharmacological activities, including antimicrobial, anti-inflammatory, antioxidant, antitumor, diuretic, and analgesic effects [19,21]. Current research on the anti-UTI activity of *Polygonum capitatum* has primarily focused on its aqueous extract [22]. In contrast, the therapeutic potential of the n-butanol extract of *Polygonum capitatum* for UTI requires further investigation. Using the disk diffusion assay, we demonstrated that the n-butanol extract of *Polygonum capitatum* (BPC) exhibits significant inhibitory activity against both the reference UPEC strain CFT073 and clinically isolated strains.

This study aims to investigate the mechanism of action of BPC against UPEC through in vitro experiments, including assessments of antibacterial activity, antibiofilm effects, anti-adhesion properties, and bacterial motility inhibition. In addition, molecular docking, based on the UHPLC-Q-Orbitrap MS/MS data, was employed to predict the active constituents within BPC targeting AKP and β-galactosidase. The findings are expected to provide insights for the development of potential therapeutic agents against urinary tract infections.

## 2. Materials and Methods

### 2.1. Bacterial Strains and Cell Culture

The UPEC strain CFT073 (ATCC^®^ 700928™) was obtained from the American Type Culture Collection (ATCC). Three clinically isolated UPEC strains were identified and provided by the Second Affiliated Hospital of Guizhou University of Traditional Chinese Medicine (Guiyang City, Guizhou Province, China). Prior to infection, all strains were cultured in LB broth at 37 °C with shaking at 180 rpm until the mid-logarithmic growth phase was reached. Bacterial suspensions were then adjusted spectrophotometrically to a concentration of 1 × 10^8^ CFU/mL for subsequent experiments.

The human urinary bladder cancer cell line T24 (ATCC^®^ HTB-4^TM^) was obtained from the ATCC. T24 cells were cultured in RPMI 1640 medium (GIBCO, Grand Island, NY, USA) supplemented with 10% (*v*/*v*) fetal bovine serum (Vivacell, Shanghai, China) and 0.5% (*v*/*v*) Penicillin-Streptomycin-Amphotericin B solution (Solarbio, Beijing, China) at 37 °C in a humidified atmosphere of 5% CO_2_.

### 2.2. Preparation of the n-Butanol Extract from Polygonum capitatum

*Polygonum capitatum* was collected from Guiyang City, Guizhou Province, China, and was botanically authenticated by Associate Professor Zehuan Wang from the College of Pharmacy, Guizhou University of Traditional Chinese Medicine. The harvested plants were air-dried, pulverized, and subsequently extracted three times (1 h each) with 70% ethanol at 60 °C using a solid-to-solvent ratio of 1:15. The filtrates were combined, concentrated under reduced pressure, and yielded a concentrated ethanolic crude extract. This ethanolic extract was then re-dissolved in distilled water and sequentially partitioned, in order of increasing polarity, using petroleum ether, ethyl acetate, and n-butanol. The resulting fractions were concentrated and lyophilized. Traditional aqueous decoction extraction of *Polygonum capitatum* indicates that its representative constituents are mainly flavonoids and phenolic acids, which is highly consistent with the frequent use of n-butanol to enrich moderately to highly polar constituents from the aqueous phase of aqueous or hydroalcoholic extracts. Therefore, for the in vitro UTI experiments in this study, BPC was selected.

### 2.3. UHPLC-Q-Orbitrap MS/MS Analysis

Fifty milligrams of BPC sample was precisely weighed into a microcentrifuge tube. One milliliter of extraction solvent (methanol:acetonitrile:water = 2:2:1, *v*/*v*/*v*) was added, followed by 20 μL of internal standard. The mixture was vortexed for 30 s. A stainless-steel bead was added, and the sample was homogenized at 45 Hz for 4 min, followed by sonication in an ice-water bath for 5 min. This homogenization–sonication cycle was repeated two to three times. After incubation at −20 °C for 1 h, the sample was centrifuged at 12,000 rpm and 4 °C for 15 min. Finally, 200 μL of the supernatant was collected for subsequent LC-MS/MS analysis.

LC-MS/MS analysis was performed on a 1290 Infinity II series UHPLC system (Agilent Technologies, Santa Clara, CA, USA) coupled to a mass spectrometer. Separation was achieved using a Waters UPLC HSS T3 column (1.8 μm, 2.1 × 100 mm). The injection volume was 1 μL. For positive ionization mode (ESI+), the mobile phase consisted of (A) 0.1% formic acid in water and (B) acetonitrile. For negative ionization mode (ESI−), the mobile phase consisted of (A) 5 mM ammonium acetate in water and (B) acetonitrile. A linear gradient elution was applied as follows: 0–1 min, 99% A; 1–8 min, 99% A to 1% A; 8–10 min, 1% A; 10–10.1 min, 1% A to 99% A; 10.1–12 min, 99% A. The flow rate was maintained at 0.5 mL/min. High-resolution mass spectrometry data were acquired in both positive and negative modes using a Thermo Q Exactive Orbitrap mass spectrometer (Thermo Fisher Scientific, Waltham, MA, USA). The instrument parameters were set as follows: full-scan resolution, 70,000; MS/MS resolution, 17,500; spray voltage, 3.8 kV (ESI+) and 3.1 kV (ESI−); mass range, m/z 70–1000; capillary temperature, 350 °C; sheath gas flow rate, 45 arbitrary units; auxiliary gas flow rate, 15 arbitrary units; collision energy, stepped at 15, 30, and 45 eV in normalized collision energy (NCE) mode. The raw mass spectrometry data were converted to mzXML format using ProteoWizard 3. Subsequent data processing, including retention time alignment, peak detection, peak picking, peak integration, and feature comparison, was conducted using the XCMS 3.6.1 software.

### 2.4. Antibacterial Activity of BPC

The antibacterial susceptibility of BPC against various UPEC strains was evaluated using the disk diffusion assay [23]. Sterile blank susceptibility disks (6 mm × 1 mm) were immersed in BPC solutions at different concentrations (100, 50 and 25 mg/mL). Diluted UPEC bacterial suspensions were spread uniformly onto LB agar plates and allowed to air-dry for 20 min. The impregnated disks were then placed equidistantly on the agar surface, with sterile water-impregnated disks serving as the control. All experiments were performed in triplicate. After incubation at 37 °C for 18–24 h, diameters of the inhibition zones (including the disk) were measured in millimeters.

### 2.5. Determination of Minimum Inhibitory Concentration (MIC) and Minimum Bactericidal Concentration (MBC)

The minimum inhibitory concentration (MIC) of BPC against various UPEC strains was determined using the broth microdilution method in 96-well microtiter plates, in accordance with the guidelines established by the Clinical and Laboratory Standards Institute (CLSI) [24]. Briefly, two-fold serial dilutions of BPC (ranging from 1 to 256 mg/mL) were prepared in LB broth in a volume of 100 µL per well. Subsequently, 100 µL of bacterial suspension was added to each well, and the plates were incubated at 37 °C for 24 h. The MIC was defined as the lowest concentration at which no visible bacterial growth was observed. To determine the minimum bactericidal concentration (MBC) of BPC against UPEC, 10 µL aliquots were taken from wells showing no visible growth (at the MIC) and from the next three higher concentration wells, and then spread onto LB agar plates [25]. After incubation at 37 °C for 24 h, the colony-forming units were counted. The MBC was defined as the lowest concentration that resulted in no observable colony growth. All experiments were performed in triplicate. The MBC/MIC ratio was determined based on established criteria in the literature [26]. A ratio of MBC/MIC ≤ 4 was interpreted as indicative of bactericidal activity, whereas a ratio > 4 was defined as bacteriostatic.

### 2.6. Time–Kill Kinetic Assays of BPC

Time–kill kinetic assays were performed to further investigate the antibacterial activity of BPC against various UPEC strains, following a previously described method with minor modifications [27]. Briefly, 5 mL of bacterial suspension (1 × 10^6^ CFU/mL) was added to 5 mL of BPC solutions at various concentrations, adjusting the final BPC concentrations to 1/4 MIC, 1/2 MIC, and MIC. The mixture was incubated at 37 °C for 24 h, with samples collected at specified time intervals (0, 4, 8, 16, and 24 h). Collected samples were serially diluted in phosphate-buffered saline (PBS), plated onto LB agar, and incubated at 37 °C for 16–18 h before colony enumeration. All experiments were performed in triplicate.

### 2.7. Anti-Biofilm Activity of BPC

#### 2.7.1. Inhibitory Effect of BPC on UPEC Biofilm Formation

The potential of BPC to inhibit the biofilm formation of various UPEC strains was assessed using the crystal violet staining method in 96-well microtiter plates [28,29]. The UPEC suspension was diluted 1:100 in LB broth to obtain a bacterial culture of approximately 1 × 10^6^ CFU/mL. Then, 100 μL of varying concentrations of BPC was added to the wells, followed by inoculation with 100 μL of the bacterial suspension to achieve final BPC concentrations equivalent to 1/4 MIC, 1/2 MIC, and MIC. Untreated bacterial suspensions served as the control. The plates were co-incubated at 37 °C for 24 and 48 h. After incubation, the medium was discarded, and the plates were gently washed three times with PBS (pH 7.4) to remove non-adherent cells. The biofilms were then fixed with methanol for 15 min. After air-drying, the plates were stained with 0.1% crystal violet (200 μL per well) for 10 min. The dye was then removed, and the plates were rinsed three times with PBS. Following drying, the bound dye was solubilized with 33% acetic acid for 15 min, and the absorbance was measured at 595 nm using a microplate reader. All experiments were performed in triplicate.

#### 2.7.2. Optical Microscopy Observation

To examine the effect of BPC on UPEC biofilm growth and development, the architecture of the biofilms was visualized using optical microscopy following crystal violet staining [30,31]. UPEC suspensions (1 × 10^8^ CFU/mL) were incubated with BPC in 24-well plates containing sterile glass coverslips for 24 h. After incubation, the culture medium was discarded, and the slides were gently washed twice with PBS. The biofilms were then stained with 0.4% (*w*/*v*) crystal violet for 10 min, after which the slides were rinsed with PBS to remove impurities and excess stain. Images of the biofilms were captured at a magnification of 400× using an Olympus CX41 microscope fitted with a digital camera.

#### 2.7.3. Effect of BPC on UPEC Cell Membrane Permeability

The effect of sub-MIC concentrations of BPC on UPEC cell membrane integrity was evaluated by measuring the leakage of intracellular components, including AKP, β-galactosidase, and total intracellular proteins [32,33,34]. Briefly, BPC was added to logarithmic-phase CFT073 cultures to achieve final concentrations of 0, 1/16 MIC, 1/8 MIC, and 1/4 MIC, followed by incubation at 37 °C for 24 h. After centrifugation, the supernatants were collected. AKP and β-galactosidase activities were measured using commercial assay kits (Jiancheng Bioengineering Institute, Nanjing, China; and Solarbio, Beijing, China, respectively) according to the manufacturers’ instructions. The leakage of total intracellular proteins was quantified using a BCA protein assay kit (Solarbio, Beijing, China).

#### 2.7.4. Molecular Docking

To further investigate the binding interactions of the compounds identified in BPC with the crystal structures of *E. coli* AKP (PDB ID: 1EW8) and β-galactosidase (PDB ID: 1JYW), molecular docking experiments were conducted [35,36]. Based on the aforementioned UHPLC-Q-Orbitrap MS/MS data, the 2D structures of the corresponding compounds were retrieved from the PubChem database (https://pubchem.ncbi.nlm.nih.gov/) to establish a compound library for BPC. The structures of the collected compounds were subsequently prepared using the LigPrep module in Schrödinger 2022 [37]. The protein structures of AKP and β-galactosidase were preprocessed by adding missing side chains and hydrogen atoms, and by removing water molecules within 5 Å of the binding sites. Both ligands and receptors were optimized using the OPLS_2005 force field.

### 2.8. Bacterial Surface Hydrophobicity Assay

The effect of BPC on UPEC surface hydrophobicity was quantified using the hydrocarbon-xylene partition assay, as previously described [38]. Briefly, CFT073 cultures were incubated with sub-MIC concentrations of BPC (1/16 MIC, 1/8 MIC, and 1/4 MIC) at 37 °C with shaking at 250 rpm for 24 h. A control culture without BPC was processed in parallel. Subsequently, 1 mL of the bacterial suspension was centrifuged at 15,000× *g* for 10 min. The pellet was washed twice with PBS and resuspended in 1 mL of PBS. Then, 250 μL of xylene was added to the bacterial suspension, and the mixture was vortexed vigorously for 30 min. The optical density (OD) at 600 nm was measured before vortexing (A_0_) and after phase separation for the aqueous phase (A_i_). The percentage of hydrophobicity was calculated using the following formula:

$$\mathrm{Percent}\;\mathrm{hydrophobicity}\;(\%H)=(A_{0}-A_{i})\times 100/A_{i}$$

### 2.9. Effect of BPC on UPEC Motility

As described previously, the effects of sub-MIC concentrations (1/2 MIC and 1/4 MIC) of BPC on UPEC swimming and swarming motility were assessed using soft agar assays [28]. A 5 μL aliquot of UPEC culture (1 × 10^8^ CFU/mL) was spot-inoculated onto swimming agar plates (containing 1% (*w*/*v*) tryptone, 0.5% (*w*/*v*) yeast extract, 0.5% (*w*/*v*) NaCl, and 0.3% (*w*/*v*) agar) and swarming agar plates (containing 1% (*w*/*v*) tryptone, 0.5% (*w*/*v*) yeast extract, 0.5% (*w*/*v*) NaCl, and 0.5% (*w*/*v*) agar). The plates were incubated at 37 °C for 24 h, after which the diameters of the motility zones were measured. All experiments were performed in triplicate.

### 2.10. Assessment of T24 Cell Viability Following BPC Treatment

The viability of T24 cells following treatment with BPC was determined using a CCK-8 assay kit (APExBIO, Houston, TX, USA). T24 cells were seeded in 96-well plates at a density of 5 × 10^3^ cells/well in 100 μL of medium and incubated for 24 h at 37 °C. After incubation, the cells were washed with PBS and treated with various final concentrations of BPC (2, 4, 8, 16, 32, 64, 128, 256, and 512 μg/mL) for 24 h. Subsequently, 10 μL of CCK-8 solution was added to each well, followed by incubation for a further 1 h. Finally, the absorbance at 450 nm was measured using a microplate reader.

### 2.11. Adhesion and Invasion Assays

The inhibitory effect of BPC on UPEC adhesion to and invasion of T24 cells was evaluated [22]. T24 cells were resuspended in antibiotic-free RPMI-1640 medium and seeded into 24-well plates at a density of 1 × 10^5^ cells per well, followed by overnight incubation to allow for cell adhesion. After washing with PBS, the cells were treated with various concentrations of BPC for 24 h prior to infection. The control group was treated with RPMI-1640 medium only. The cells were then infected with UPEC at a multiplicity of infection (MOI) of 100:1 for 2 h. Subsequently, the medium was discarded, and the cells were washed three times with PBS to remove non-adherent bacteria. For the adhesion assay, 200 μL of 0.25% Triton X-100 was added to lyse the cells for 15 min. The eluted bacteria were serially diluted and plated onto LB agar plates. After incubation at 37 °C for 18 h, bacterial colonies were counted. The relative adhesion rate was calculated as follows: (CFU of each group/CFU in control) × 100%. For the invasion assay, after infection, the cells were treated with 100 μg/mL gentamicin for 30 min to kill extracellular bacteria. After washing with PBS, the cells were lysed using the method described for the adhesion assay. The colonies were then counted to calculate the relative invasion rate.

### 2.12. Statistical Analysis

Statistical analyses were performed using IBM SPSS Statistics for Windows, version 27.0 (IBM Corp., Armonk, NY, USA). Data are presented as the mean ± standard deviation from three independent experiments. Normality of the data was assessed using the Shapiro–Wilk test, and homogeneity of variance was evaluated with Levene’s test. If the data met the assumptions of normality and homogeneity of variance, one-way analysis of variance (ANOVA) followed by Fisher’s Least Significant Difference (LSD) post hoc test was employed to compare group differences. When these assumptions were violated, Dunn’s test was applied. A *p*-value ≤ 0.05 was considered statistically significant.

## 3. Results and Discussion

### 3.1. Phytochemical Profiling of BPC by UHPLC-Q-Orbitrap MS/MS

**Figure 1:**
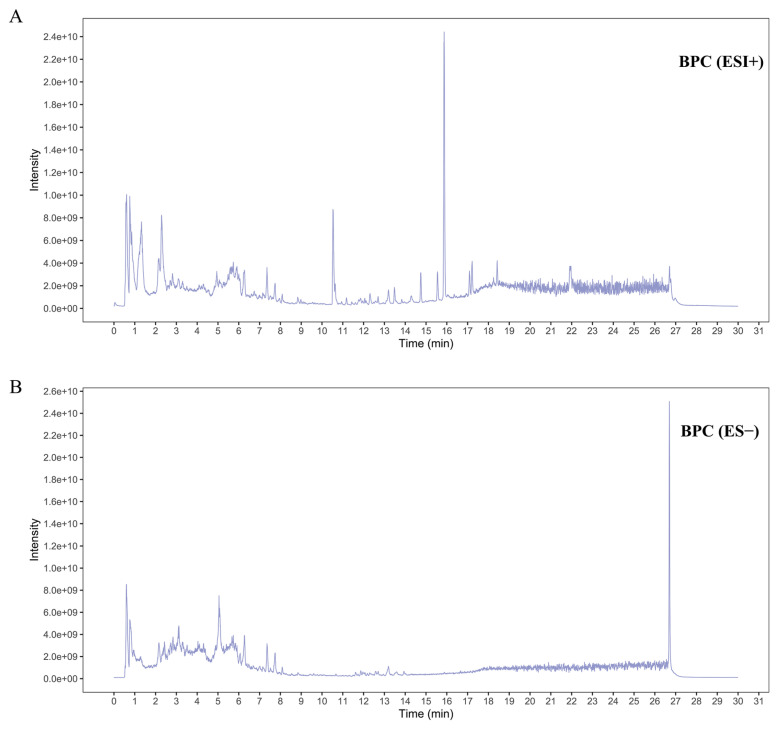
Total ion chromatogram of BPC obtained by UHPLC-Q-Orbitrap MS/MS. (**A**) Positive ion mode. (**B**) Negative ion mode.

The chemical composition of BPC was analyzed using UHPLC-Q-Orbitrap MS/MS in both positive and negative ionization modes (Figure 1), and constituents were tentatively identified. A total of 32 compounds were putatively characterized in BPC, predominantly flavonoids, along with smaller amounts of phenolic acids, saccharides, and other compound classes. These compounds are listed in Supplementary Table S1, detailing their compound names, molecular formulas, retention times, and median mass-to-charge ratios (mzmed).

### 3.2. Antibacterial Activity of BPC Against UPEC

As shown in Table 1 and Supplementary Figure S1, BPC exhibited concentration-dependent antibacterial activity against the UPEC reference strain CFT073 and three clinically isolated UPEC strains. Distinct inhibition zones were observed at the tested BPC concentrations, with diameters ranging from 7.0 to 12.6 mm. Comparative analysis of the MIC and MBC values revealed that BPC possessed stronger antibacterial efficacy against two clinical isolates (UPEC01 and UPEC03) than against the reference strain. The MIC and MBC for CFT073 were 16 mg/mL and 128 mg/mL, respectively. In contrast, the MIC and MBC for the clinical isolates ranged from 4 mg/mL to 128 mg/mL. Notably, BPC displayed a bacteriostatic effect (MBC/MIC > 4) against strains CFT073 and UPEC02, while it exhibited a bactericidal effect (MBC/MIC ≤ 4) against strains UPEC01 and UPEC03. Given the potent inhibitory effects of BPC on UPEC, we further investigated its potential antibacterial mechanisms.

**Figure 2:**
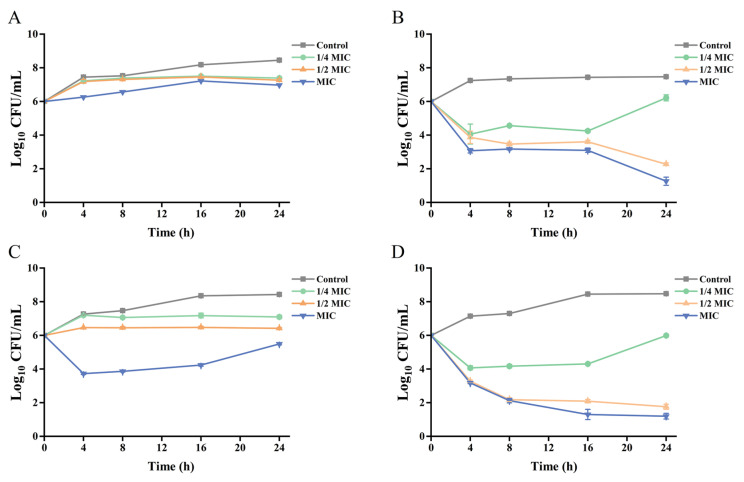
Time–kill kinetic assays of BPC against various UPEC strains: CFT073 (**A**), UPEC01 (**B**), UPEC02 (**C**), and UPEC03 (**D**). Bars represent the mean ± standard deviation (SD) of independent experiments conducted in triplicate.

The bactericidal efficacy of BPC at different concentrations (1/4 MIC, 1/2 MIC, and MIC) against the UPEC strains (CFT073, UPEC01, UPEC02, and UPEC03) was further evaluated using time–kill kinetic assays, with the results presented in Figure 2. BPC suppressed the growth of all tested strains in a time-dependent manner, with its effects demonstrating both concentration-dependent and strain-dependent characteristics. Notably, consistent with the MIC values above-mentioned, BPC exhibited more potent antibacterial activity against the clinically isolated strains. As shown in Figure 2A, BPC at its MIC (16 mg/mL) markedly inhibited the growth of CFT073 from 4 to 24 h. In contrast, as depicted in Figure 2B–D, all tested concentrations of BPC inhibited the growth of the clinical UPEC isolates throughout the entire incubation period. Particularly against UPEC01 and UPEC03, the inhibitory effect was the most pronounced. The lowest viable bacterial counts were observed at 24 h for both the MIC (4 mg/mL) and 1/2 MIC (2 mg/mL) concentrations.

### 3.3. BPC Inhibits UPEC Biofilm Formation

**Figure 3:**
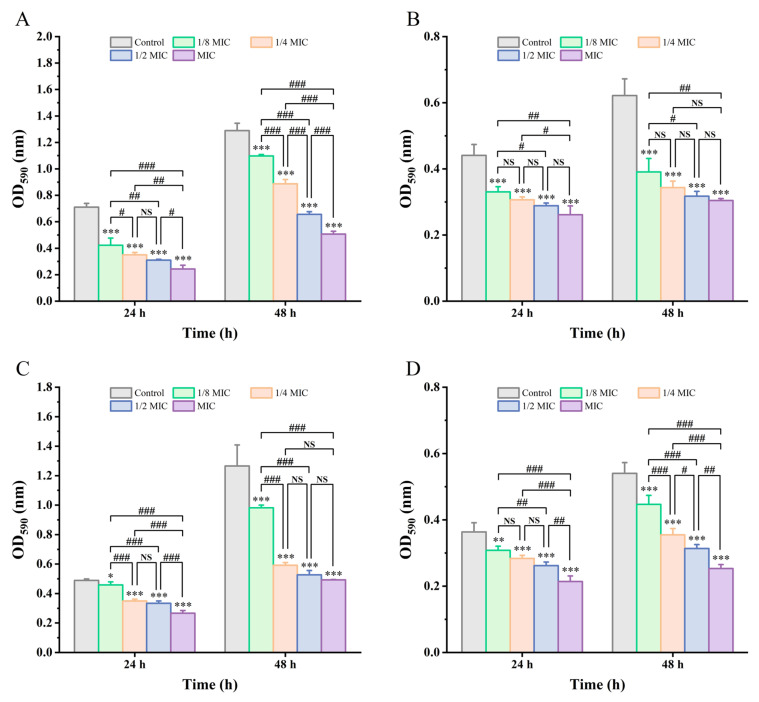
Effects of BPC treatment for 24 h and 48 h on biofilms of various UPEC strains. (**A**) CFT073 group; (**B**) UPEC01 group; (**C**) UPEC02 group; (**D**) UPEC03 group. Data are presented as the mean ± standard deviation (SD) from at least three independent experiments. NS (not statistically significant), * *p* < 0.05, ** *p* < 0.01, and *** *p* < 0.001 versus the control group. # *p* < 0.05, ## *p* < 0.01 and ### *p* < 0.001 were used for comparisons with the indicated treatment groups.

Biofilm formation is a key virulence factor that enhances pathogenicity and facilitates persistent infection. The anti-biofilm effects of BPC against various UPEC strains were evaluated using the crystal violet staining assay. As shown in Figure 3, BPC significantly inhibited biofilm formation in all UPEC strains at both 24 and 48 h in a concentration- and time-dependent manner. Although strain-dependent variations in susceptibility to BPC were observed, significant biofilm inhibition was consistently achieved at higher concentrations and strains, particularly UPEC01 and UPEC03. The biofilm inhibition rates following treatment with different concentrations of BPC against CFT073, UPEC01, UPEC02, and UPEC03 were 40.64–65.82% and 14.86–60.68%; 25.04–40.69% and 37.12–51.06%; 6.23–45.52% and 22.42–61.07%; and 15.31–41.14% and 17.33–53.16% at 24 and 48 h, respectively. These results suggest that BPC effectively disrupts UPEC biofilms, thereby contributing to its antibacterial efficacy.

**Figure 4:**
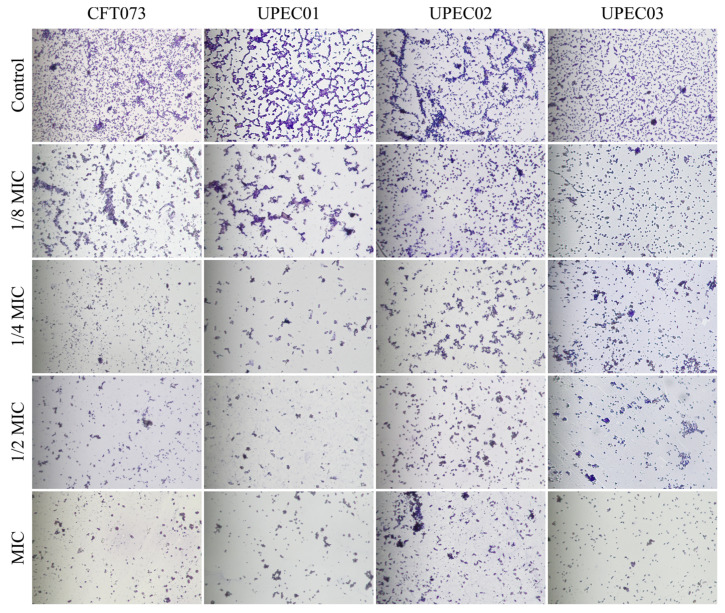
Representative images of crystal violet-stained UPEC biofilms following treatment with increasing concentrations of BPC (biofilm images were captured using a 400× magnification).

As shown in Figure 4, consistent with the crystal violet staining results described above, BPC reduced biofilm formation across different UPEC strains. In the control groups of various UPEC strains, biofilms exhibited dense, reticular, and clustered structures, indicating robust biofilm development. In contrast, the structural integrity of biofilms was markedly disrupted following BPC treatment, with the effect being both concentration-dependent and evident across all strains examined. These findings indicate that BPC effectively inhibits UPEC biofilm formation and disrupts maturation.

### 3.4. Sub-MIC Concentrations of BPC Compromise Membrane Integrity in UPEC Strain CFT073

**Figure 5:**
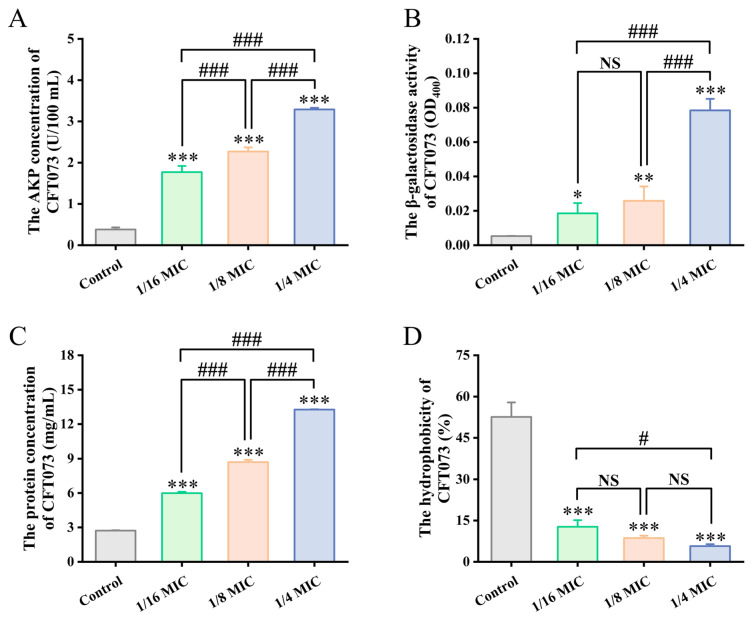
Effects of BPC treatment at different concentrations on AKP (**A**) and β-galactosidase activity (**B**), intracellular protein concentration (**C**), and cell surface hydrophobicity of UPEC strain CFT073 (**D**). Data are presented as the mean ± standard deviation (SD) from at least three independent experiments. NS (not statistically significant), * *p* < 0.05, ** *p* < 0.01 and *** *p* < 0.001 versus the control group. # *p* < 0.05 and ### *p* < 0.001 were used for comparisons between study groups.

Having observed its potent inhibitory effect on UPEC biofilms, we next investigated the effect of sub-MIC BPC concentrations on cell membrane permeability in UPEC strain CFT073, specifically measuring the leakage of AKP, β-galactosidase, and total intracellular proteins, with the results presented in Figure 5A–C.

AKP, an enzyme located in the periplasmic space between the cell wall and cell membrane, is typically detectable in the extracellular milieu only when the bacterial cell wall is compromised (e.g., due to cell deformation, rupture, or death). Consequently, its extracellular activity serves as an indicator of cell wall integrity. As shown in Figure 5A, extracellular AKP activity in UPEC strain CFT073 was increased with higher concentrations of BPC. Compared with the control group, treatment with BPC at 1/16 MIC, 1/8 MIC, and 1/4 MIC for the same incubation period increased extracellular AKP activity approximately 4.66-fold, 5.98-fold, and 8.66-fold, respectively. These results indicate that BPC markedly increases cell wall permeability, leading to the leakage of intracellular AKP and consequently elevating extracellular AKP activity in UPEC.

β-Galactosidase is a cytoplasmic enzyme located adjacent to the bacterial inner membrane. Measurement of its activity in the culture supernatant can be used to assess the effect of antimicrobial agents on bacterial membrane integrity. BPC enhanced the leakage of β-galactosidase of CFT073 in a concentration-dependent manner. As shown in Figure 5B, BPC at 1/16 MIC, 1/8 MIC, and 1/4 MIC increased the extracellular β-galactosidase activity approximately 3.48-fold, 4.86-fold, and 14.80-fold, respectively, levels that were significantly higher than those in the control group.

Consistent with the findings for AKP and β-galactosidase, extracellular total protein levels also increased with BPC concentration in a concentration-dependent manner (Figure 5C). Compared with the control group, the release of extracellular proteins was enhanced approximately 2.20-fold, 3.20-fold, and 4.88-fold, respectively.

### 3.5. Sub-MIC Concentrations of BPC Reduce the Surface Hydrophobicity of UPEC Strain CFT073

To further elucidate the mechanism by which BPC inhibits UPEC biofilm formation, its effect on the cell surface hydrophobicity of CFT073 was evaluated. As shown in Figure 5D, BPC at 1/4 MIC, 1/8 MIC, and 1/16 MIC reduced the cell surface hydrophobicity of CFT073 by 39.87%, 43.98%, and 46.89%, respectively, compared to the control. Therefore, BPC may inhibit biofilm formation by reducing bacterial cell surface hydrophobicity.

### 3.6. Molecular Interactions of AKP, and β-Galactosidase with Chemical Constituents of BPC

**Figure 6:**
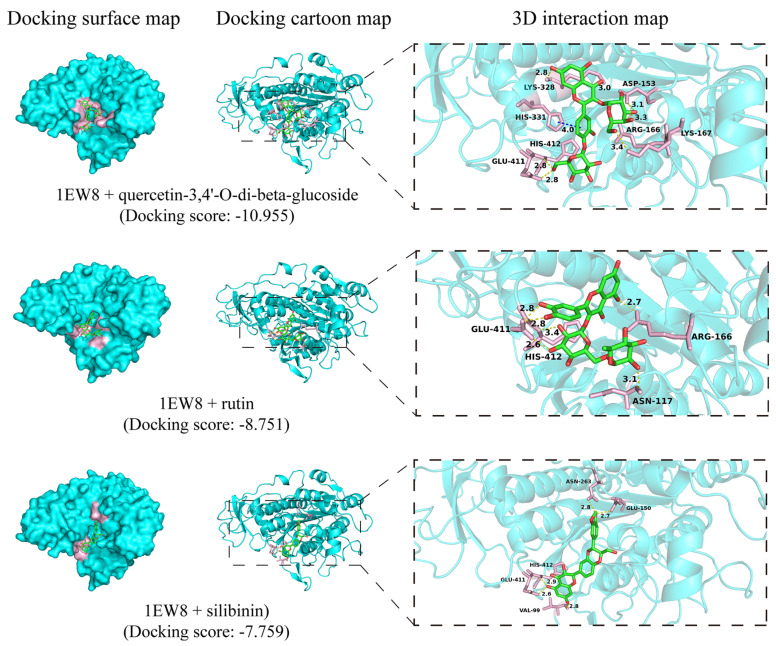
Molecular docking of representative BPC constituents with AKP (PDB ID: 1EW8). The binding modes and interactions of the top-scoring compounds are illustrated.

**Figure 7:**
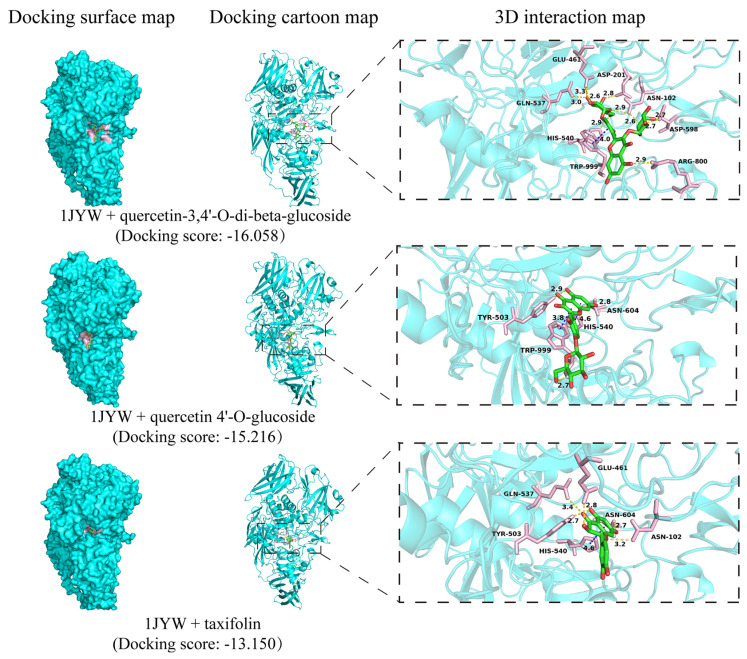
Molecular docking of representative BPC constituents with β-galactosidase (PDB ID: 1JYW). The predicted binding conformations and key interacting residues are shown.

To further explore the binding affinities and specific molecular interactions between the compounds identified in BPC and the target enzymes, AKP and β-galactosidase, a molecular docking analysis was performed, with the results presented in Figure 6 and Figure 7. As shown in Figure 6, molecular docking analysis revealed that flavonoid compounds exhibited favorable binding affinity to AKP (PDB ID: 1EW8). The top-ranked docking poses were observed for quercetin-3,4′-O-di-beta-glucoside, rutin, and silibinin. Their binding was primarily stabilized by hydrogen bonds with residues including ARG-166, GLU-411, HIS-412, and ARG-166, yielding docking scores ranging from −10.955 to −7.759. β-Galactosidase (PDB ID: 1JYW) showed high-affinity binding to quercetin-3,4′-O-di-beta-glucoside, quercetin-4′-O-glucoside, and taxifolin (Figure 7), with corresponding docking scores of −16.058, −15.216, and −13.150, respectively. These interactions were predominantly mediated by hydrogen bonds with residues HIS-540, ASN-102, GLN-537, and GLU-461. Notably, quercetin-3,4′-O-di-beta-glucoside yielded the highest predicted docking scores for both target enzymes, AKP, and β-galactosidase.

### 3.7. BPC Inhibits Swimming and Swarming Motility in UPEC

**Figure 8:**
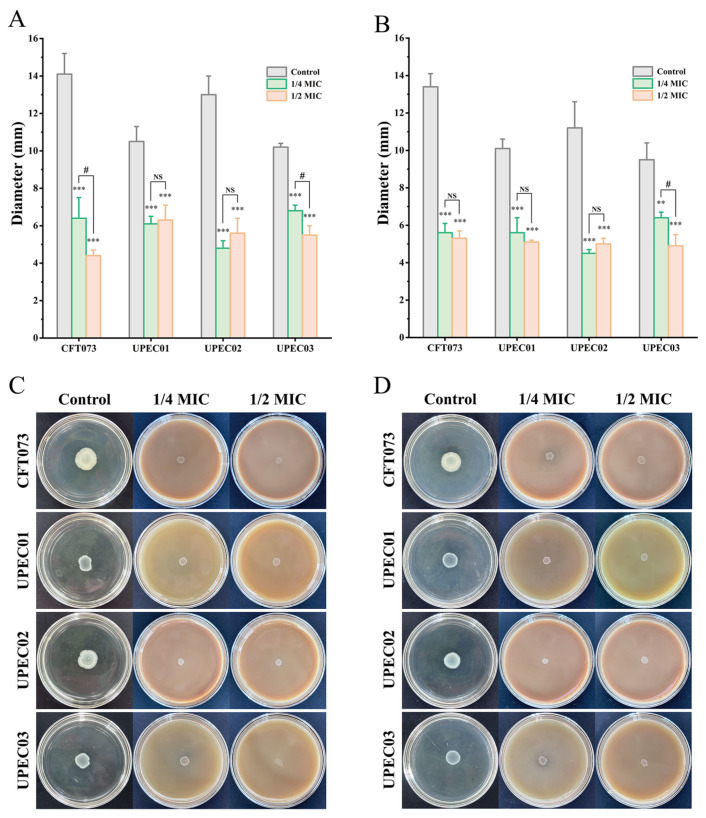
Effect of sub-MIC concentrations of BPC on UPEC motility. Swimming (**A**,**C**) and swarming (**B**,**D**) motility were assessed following treatment with BPC at 1/4 × MIC and 1/2 × MIC. Data are presented as the mean ± standard deviation (SD) from at least three independent experiments. NS (not statistically significant), ** *p* < 0.01 and *** *p* < 0.001 versus the control group. # *p* < 0.05 for comparisons between indicated study groups.

Bacterial motility is a critical determinant of UPEC colonization, contributing to intra-host movement, surface attachment, and subsequent biofilm formation associated with persistent infections. Accordingly, swarming and swimming motilities were assessed using soft agar assays. As shown in Figure 8, treatment with sub-MIC concentrations of BPC markedly reduced both swarming and swimming motilities across all tested UPEC strains compared to the untreated control. Specifically, 1/4 MIC and 1/2 MIC of BPC resulted in swimming motility inhibition rates of 54.61% and 68.79% for CFT073, 41.90% and 40.00% for UPEC01, 63.08% and 56.92% for UPEC02, and 33.33% and 46.08% for UPEC03. Similarly, swarming motility was inhibited by 58.21% and 60.45% for CFT073, 44.55% and 49.50% for UPEC01, 59.82% and 55.36% for UPEC02, and 32.63% and 48.42% for UPEC03, respectively. These results indicate that BPC effectively restrains UPEC swimming and swarming motility, which may consequently impair its ability to form biofilms.

### 3.8. BPC Inhibits UPEC Adhesion to and Invasion of T24 Cells

**Figure 9:**
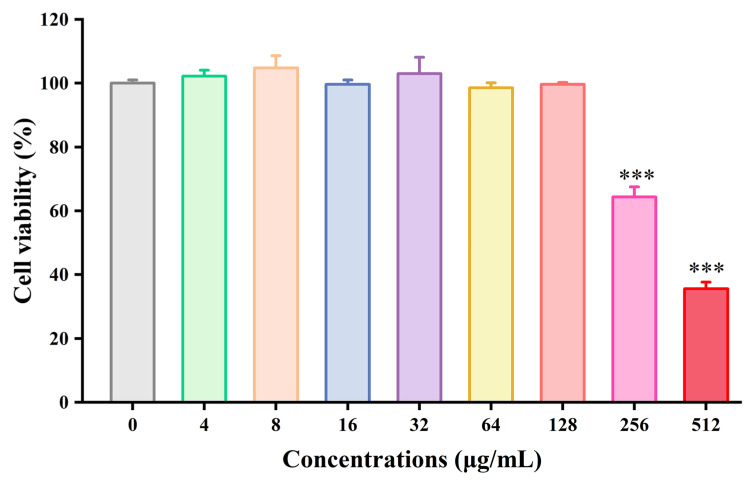
Effect of BPC (0–512 µg/mL) on T24 cell viability (CCK-8 assay) after 24-h incubation. Data are presented as the mean ± standard deviation (SD) from at least three independent experiments. *** *p* < 0.001 versus the control group.

Prior to assessing the anti-adhesive and anti-invasive effects of BPC, its cytotoxicity against T24 human bladder epithelial cells was evaluated using a CCK-8 assay to determine non-cytotoxic concentrations for subsequent experiments. As shown in Figure 9, treatment of T24 cells with varying concentrations of BPC (ranging from 4 to 512 μg/mL) for 24 h revealed that the concentrations exceeding 128 μg/mL caused the most significant cell damage (*p* < 0.001) compared to the control group. BPC concentrations below 128 μg/mL showed no significant toxicity, and cell viability was statistically indistinguishable from the control group. Consequently, subsequent experiments utilized BPC concentrations at or below this threshold.

**Figure 10:**
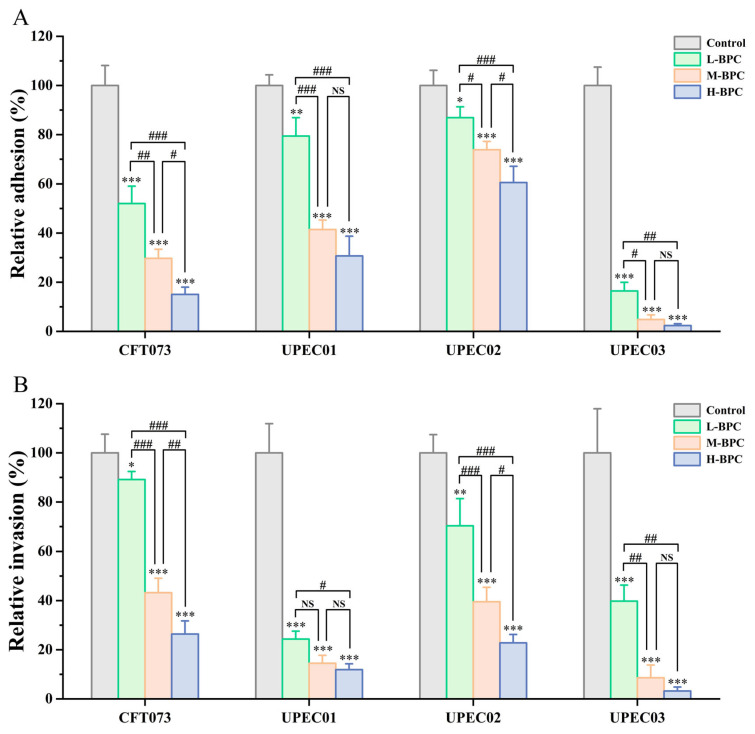
Inhibitory effect of BPC on UPEC adhesion to and invasion of T24 cells. T24 cells were treated with low (L-BPC, 32 μg/mL), medium (M-BPC, 64 μg/mL), and high (H-BPC, 128 μg/mL) concentrations of BPC prior to infection with UPEC strains. Adhesion (**A**) and invasion (**B**) were subsequently quantified. Data are presented as the mean ± standard deviation (SD) from at least three independent experiments. NS (not statistically significant), * *p* < 0.05, ** *p* < 0.01 and *** *p* < 0.001 versus the control group. # *p* < 0.05, ## *p* < 0.01 and ### *p* < 0.001 were used for comparisons between study groups.

Adhesion to and invasion of uroepithelial cells by UPEC represent the initial and critical steps in the pathogenesis of UTI, which are essential for the colonization of host uroepithelial tissues. Based on the aforementioned cytotoxicity results, this study investigated the effects of low (32 μg/mL), medium (64 μg/mL), and high (128 μg/mL) doses of BPC on the adhesion and invasion of various UPEC strains using a T24 cell infection model. As shown in Figure 10, while different UPEC strains rapidly adhered to and invaded T24 cells, BPC significantly reduced adhesion and invasion capabilities in a concentration-dependent manner. In the adhesion assay (Figure 10A), various concentrations of BPC exhibited potent inhibitory effects on both the reference strain CFT073 and the clinically isolated strain UPEC03 (*p* < 0.001). Compared to the untreated control, the high-dose BPC (H-BPC) group reduced the relative adhesion rates of UPEC strains CFT073, UPEC01, UPEC02, and UPEC03 to 15.03%, 30.70%, 60.54%, and 2.38%, respectively (*p* < 0.001). A similar inhibitory trend was observed in the invasion assay (Figure 10B). All tested concentrations of BPC reduced UPEC invasion into T24 cells, with the strongest suppression seen against the clinically isolated strains UPEC01 and UPEC03.

## 4. Discussion

Preliminary screening using the disk diffusion assay revealed that BPC exhibited antibacterial activity against both reference and clinically isolated UPEC strains in a concentration-dependent manner. Notably, at a concentration of 100 mg/mL, BPC produced an inhibition zone of up to 12.6 mm in diameter (Table 1 and Figure S1). Further analysis of MIC, MBC, and MBC/MIC ratios indicated that BPC exerted a bactericidal effect against the UPEC strains, rather than merely inhibiting their growth (Table 1). This finding was further corroborated by time–kill curve assays (Figure 2), which also revealed that the clinical isolates were more susceptible to BPC than the reference strain. Given the importance of this property for controlling acute infections and preventing the emergence of bacterial resistance, the effects of sub-MIC and MIC concentrations of BPC on various functional and phenotypic characteristics of the UPEC were subsequently investigated.

In the pathogenesis of UPEC-induced UTI, the ability to form biofilms is a central factor mediating infection establishment, persistence, and recurrence of infection [10]. Our quantitative crystal violet staining and optical microscopy observations demonstrated that the inhibitory effect of BPC on UPEC biofilm formation was both concentration- and time-dependent. This suggests that the anti-biofilm activity of BPC is not merely transient but is sustained or even enhanced over time. Consistent with previous studies, extracts from numerous plant species, such as *Solidago virgaurea* and aqueous or methanol extracts of *Aloe vera*, have been shown to significantly inhibit UPEC biofilm formation [39,40]. Furthermore, BPC exerted inhibitory effects against all tested UPEC strains, indicating a broad-spectrum anti-biofilm activity.

Damage to the bacterial cell membrane, leading to cytoplasmic leakage, can severely disrupt cellular metabolism [41]. AKP, β-galactosidase, and intracellular proteins are therefore commonly used as indicators of cell membrane damage [32,33]. We hypothesized that BPC may inhibit and kill UPEC by damaging the bacterial cell membrane. In this study (Figure 5), treatment with BPC induced significant increases in the membrane permeability of UPEC strain CFT073, resulting in enhanced leakage of intracellular components, including AKP, β-galactosidase, and total intracellular proteins. These results strongly suggest that BPC disrupts the structural integrity of the UPEC cell membrane, leading to the loss of intracellular contents and ultimately culminating in cell lysis and death. This mechanism, which shares similarities with the action of certain antimicrobial peptides or surfactants, may reduce the propensity for inducing conventional antibiotic resistance [42]. To identify the active constituents in BPC responsible for modulating AKP and β-galactosidase, 32 compounds were putatively characterized using UHPLC-Q-Orbitrap MS/MS. The potential active components and their mechanisms of action were further explored through molecular docking. Molecular docking analysis revealed that AKP exhibited strong binding affinity with quercetin-3,4′-O-di-beta-glucoside, rutin, and silibinin in BPC, while β-galactosidase showed significant molecular interactions with quercetin-3,4′-O-di-beta-glucoside, quercetin-4′-O-glucoside, and taxifolin. These computational findings support their potential as inhibitors of AKP and β-galactosidase, which is consistent with the observed cytoplasmic leakage in UPEC upon extract treatment. Together, these results suggest that specific compounds in BPC act as inhibitors by interacting with AKP or β-galactosidase in the UPEC cell membrane, thereby disrupting membrane integrity, increasing permeability, and inducing leakage of these enzymes—ultimately impairing the ability of UPEC to form biofilms. Cell surface hydrophobicity facilitates the adhesion of uropathogens to both biotic and abiotic surfaces [5]. Treatment with BPC significantly reduced bacterial surface hydrophobicity, providing an additional mechanism by which BPC may impair UPEC colonization and biofilm formation

Motility and adhesion are critical for the virulence and pathogenicity of UPEC [4,12]. Treatment with sub-MIC concentrations of BPC markedly inhibited both forms of motility across various UPEC strains, suggesting that it effectively interferes with the synthesis or function of flagella, the bacterial organelles responsible for locomotion. Previous studies have demonstrated that the aqueous extract of *Polygonum capitatum* significantly inhibits UPEC motility, a finding consistent with our observations in this study (Figure 8) [22]. Adhesion and invasion are critical factors for UPEC to successfully establish an infection [43]. Therefore, reducing UPEC adhesion and invasion represents a promising strategy for preventing or treating urinary tract infection. In this study (Figure 10), BPC treatment significantly reduced the bacterial load on T24 cells in a dose-dependent manner, indicating that the adhesive and invasive capabilities of diverse UPEC strains were substantially impaired by BPC. The interconnectedness of UPEC motility, adhesion, invasion, and biofilm formation underscores the multi-target mode of action of BPC, which provides a distinct advantage in combating the complex pathogenicity of UPEC. For instance, in biofilm-associated or recurrent UTIs, the reduction in adhesion and biofilm burden may enhance host clearance and improve antibiotic accessibility, positioning BPC as a potential adjunctive strategy to antibiotic therapy.

Although this study provides some important findings, several limitations exist. Further validation can be performed in UTI murine models to determine the pharmacokinetic profile and in vivo efficacy of BPC. Additionally, exploring potential synergistic interactions between BPC and commonly used clinical antibiotics could facilitate enhanced therapeutic efficacy while enabling dose reduction and mitigating the risk of resistance development.

## 5. Conclusions

This study elucidates the antibacterial activity and anti-biofilm mechanism of the n-butanol extract from *Polygonum capitatum* against both the reference UPEC strain CFT073 and clinically isolated UPEC strains. Furthermore, biofilm formation through multiple pathways was inhibited by increasing cell membrane permeability (evidenced by leakage of intracellular AKP, β-galactosidase, and proteins), reducing bacterial surface hydrophobicity, suppressing motility, and impairing adhesion and invasion capabilities. A total of 32 compounds in BPC were identified by UHPLC-Q-Orbitrap MS/MS. Subsequent molecular docking studies revealed that several flavonoid compounds, such as quercetin-3,4′-O-di-beta-glucoside, exhibit strong binding affinity to AKP and β-galactosidase, suggesting their potential as effective inhibitors of these enzymes in UPEC. These findings collectively support BPC’s promise as a novel therapeutic or adjunctive agent for urinary tract infections, particularly against stubborn biofilm-associated infections.

## Figures and Tables

**Table 1:** MIC, MBC, MBC/MIC ratio, and inhibition zone diameters of BPC against various UPEC strains.

Strains	MIC (mg/mL)	MBC (mg/mL)	MBC/MIC	Inhibition Zone Diameters (mm)		
				25 mg/mL	50 mg/mL	100 mg/mL
CFT073	16	128	8 (−)	7.8 ± 0.3	9.5 ± 0.7	12.6 ± 1.2
UPEC01	4	16	4 (+)	—	7.4 ± 0.3	9.8 ± 0.3
UPEC02	16	128	8 (−)	7.0 ± 0.6	9.2 ± 1.3	11.3 ± 0.9
UPEC03	4	8	2 (+)	—	7.5 ± 0.5	9.4 ± 1.2

For the MBC/MIC ratio, (+) bactericidal; (−) bacteriostatic.

## Data Availability

The original contributions presented in this study are included in the article/Supplementary Materials. Further inquiries can be directed to the corresponding authors.

## Supplementary Materials

The following supporting information can be downloaded at: https://www.mdpi.com/article/10.3390/cimb48030265/s1.
