# Supplementary material for: n-Butanol Extract of Polygonum capitatum Targets Biofilm Formation, Motility, and Adhesion Attenuation to Combat Uropathogenic Escherichia coli
Source: Curr Issues Mol Biol. 2026 Mar 2;48(3):265. doi: 10.3390/cimb48030265 (PMC13025368; doi:10.3390/cimb48030265)
Supplement: Supplementary file 1 [file cimb-48-00265-s001.zip › cimb-4153147-supplementary.pdf]

Supplementary Materials

n-Butanol Extract of *Polygonum capitatum* Targets Biofilm Formation, Motility, and Adhesion Attenuation to Combat Uropathogenic *Escherichia coli*

Derong Zeng [1,†], Yan Zhang [1,†,*], Jingjing Guo [2], Jiahua Yu [1], Shuai Dou [1], Yuqi Yang [3], Xiang Yu [1], Yongqiang Zhou [1], Juan Xue [1], Zehuan Wang [1] and Wude Yang [1,*]

[1] College of Pharmacy, Guizhou University of Traditional Chinese Medicine, Guiyang 550025, China
[2] Centre in Artificial Intelligence Driven Drug Discovery, Faculty of Applied Sciences, Macao Polytechnic University, Macao, China
[3] School of Basic Medicine, Guizhou University of Traditional Chinese Medicine, Guiyang China
* Correspondence: zhangyan0003@gzy.edu.cn (Y.Z.); ywd_680708@sina.com (W.Y.)
† These authors contributed equally to this work.

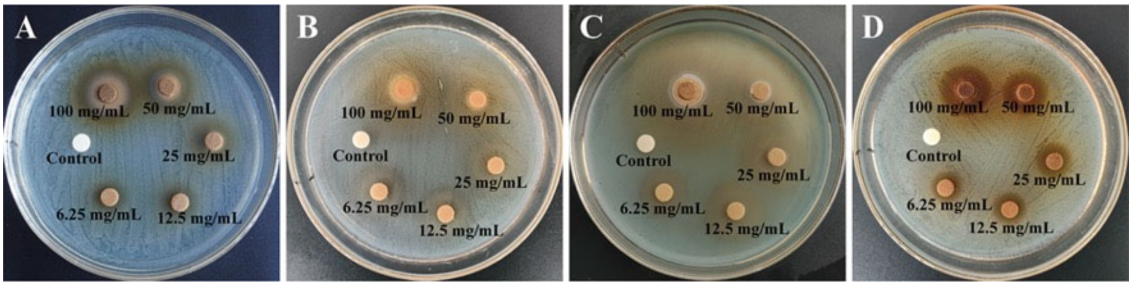

Figure S1. Inhibition zone diameters of BPC against various UPEC strains. CFT073 (**A**), UPEC01 (**B**), UPEC02 (**C**), and UPEC03 (**D**).

Table S1. Chemical profiling of BPC using UHPLC-Q-Orbitrap MS/MS in positive and negative ionization modes.

No.	Compound name	Formula	RT (min)	Error (ppm)	Adduct	Mzmed
1	Vanillic acid	$C_8H_8O_4$	0.63	1.0313	[M-H]-	167.0348
2	4-Glucogallic acid	$C_{13}H_{16}O_{10}$	0.82	0.3249	[M+H]+	333.0809
3	1,2-Digalloyl-beta-D-glucopyranose	$C_{20}H_{20}O_{14}$	1.30	0.5889	[M+H]+	485.0923
4	Sibiricose A5	$C_{22}H_{30}O_{14}$	2.42	0.4899	[M-H]-	517.1557
5	Salidroside	$C_{14}H_{20}O_7$	2.56	0.0630	[M+NH4]+	318.1540
6	1,6-Digalloyl-beta-D-glucopyranose	$C_{20}H_{20}O_{14}$	2.68	0.2555	[M-H]-	483.0771
7	Gardenoside	$C_{17}H_{24}O_{11}$	3.58	0.1034	[M-H]-	403.1240
8	Geniposide	$C_{17}H_{24}O_{10}$	3.65	0.2796	[M-H]-	387.1289
9	Quinic acid	$C_7H_{12}O_6$	3.99	1.0865	[M-H]-	191.0558
10	Quercetin-3,4'-O-di-beta-glucoside	$C_{27}H_{30}O_{17}$	4.55	1.4265	[M+H]+	627.1531
11	Luteolin-4'-O-glucoside	$C_{21}H_{20}O_{11}$	4.67	0.8481	[M+H]+	449.1074
12	Procyanidin C1	$C_{45}H_{38}O_{18}$	5.18	3.1924	[M-H]-	865.1912
13	Quercetin-4'-O-glucoside	$C_{21}H_{20}O_{12}$	5.22	1.2423	[M+H]+	465.1026
14	Kaempferol-3-O-hexoxyl-hexoside	$C_{27}H_{30}O_{16}$	5.36	1.0064	[M+H]+	611.1594
15	Rutin	$C_{27}H_{30}O_{16}$	5.50	0.6291	[M-H]-	609.1446
16	Taxifolin	$C_{15}H_{12}O_7$	5.55	1.4769	[M-H]-	303.0506
17	Eriodictyol-7-O-glucoside	$C_{21}H_{22}O_{11}$	5.69	0.7664	[M-H]-	449.1073
18	lLuteolin	$C_{15}H_{10}O6$	5.73	0.0525	[M-H]-	285.0400
19	Mahaleboside	$C1_5H_{16}O_8$	5.98	0.6294	[M+H]+	325.0912
20	Gossypetin	$C_{15}H_{10}O_8$	5.98	1.1356	[M-H]-	317.0304
21	Silibinin	$C_{25}H_{22}O_{10}$	6.03	2.0868	[M-H]-	481.1150
22	Phlorizin	$C_{21}H_{24}O_{10}$	6.19	0.1098	[M+H]+	437.1440
23	Kaempferol 7-O-glucoside	$C_{21}H_{20}O_{11}$	6.76	0.7687	[M-H]-	447.0923
24	Quercetin-3-O-glucoside	$C_{21}H_{20}O_{12}$	6.95	0.6934	[M-H]-	463.0873
25	Salicylic acid	$C_7H_6O_3$	7.02	2.3902	[M-H]-	137.0243
26	Phloretin	$C_{15}H_{14}O_5$	7.18	1.2529	[M+H]+	275.0907
27	Picein	$C_{14}H_{18}O_7$	7.24	1.4656	[M-H]-	297.1006
28	Quercetin	$C1_5H_{10}O_7$	7.50	0.0319	[M-H]-	301.0350
29	Kaempferol	$C_{15}H_{10}O_6$	8.46	0.5628	[M-H]-	285.0402
30	Isorhamnetin	$C_{16}H_{12}O_7$	8.68	0.3073	[M-H]-	315.0511
31	Gallic acid	$C_7H_6O_5$	9.03	0.0558	[M-H]-	169.0140
32	Kukoamine B	$C_{28}H_{42}N_4O_6$	14.30	0.4158	[M-H]-	529.3038
